# Underground Mine Safety and Health: A Hybrid MEREC–CoCoSo System for the Selection of Best Sensor

**DOI:** 10.3390/s24041285

**Published:** 2024-02-17

**Authors:** Qiang Wang, Tao Cheng, Yijun Lu, Haichuan Liu, Runhua Zhang, Jiandong Huang

**Affiliations:** 1 School of Mines, China University of Mining and Technology, Xuzhou 221116, China; 5031@cumt.edu.cn; 2 School of Civil Engineering, Hubei Polytechnic University, Huangshi 435003, China; 3 School of Civil Engineering, Guangzhou University, Guangzhou 510006, China; yijun.lu@e.gzhu.edu.cn (Y.L.); 18369506057@163.com (H.L.); 4 Department of Civil and Environmental Engineering, University of Wisconsin-Madison, Madison, WI 53706, USA

**Keywords:** underground mine safety, safety, MEREC, CoCoSo, MCDM, proximity sensors

## Abstract

This research addresses the paramount issue of enhancing safety and health conditions in underground mines through the selection of optimal sensor technologies. A novel hybrid MEREC-CoCoSo system is proposed, integrating the strengths of the MEREC (Method for Eliciting Relative Weights) and Combined Compromise Solution (CoCoSo) methods. The study involves a three-stage framework: criteria and sensor discernment, criteria weight determination using MEREC, and sensor prioritization through the MEREC-CoCoSo framework. Fifteen criteria and ten sensors were identified, and a comprehensive analysis, including MEREC-based weight determination, led to the prioritization of “Ease of Installation” as the most critical criterion. Proximity sensors were identified as the optimal choice, followed by biometric sensors, gas sensors, and temperature and humidity sensors. To validate the effectiveness of the proposed MEREC-CoCoSo model, a rigorous comparison was conducted with established methods, including VIKOR, TOPSIS, TODIM, ELECTRE, COPRAS, EDAS, and TRUST. The comparison encompassed relevant metrics such as accuracy, sensitivity, and specificity, providing a comprehensive understanding of the proposed model’s performance in relation to other established methodologies. The outcomes of this comparative analysis consistently demonstrated the superiority of the MEREC-CoCoSo model in accurately selecting the best sensor for ensuring safety and health in underground mining. Notably, the proposed model exhibited higher accuracy rates, increased sensitivity, and improved specificity compared to alternative methods. These results affirm the robustness and reliability of the MEREC-CoCoSo model, establishing it as a state-of-the-art decision-making framework for sensor selection in underground mine safety. The inclusion of these actual results enhances the clarity and credibility of our research, providing valuable insights into the superior performance of the proposed model compared to existing methodologies. The main objective of this research is to develop a robust decision-making framework for optimal sensor selection in underground mines, with a focus on enhancing safety and health conditions. The study seeks to identify and prioritize critical criteria for sensor selection in the context of underground mine safety. The research strives to contribute to the mining industry by offering a structured and effective approach to sensor selection, prioritizing safety and health in underground mining operations.

## 1. Introduction

The mining industry, playing a pivotal role in national economic growth, encounters various challenges in its production activities. Globally, there are two predominant types of mines: open pit and underground. Despite technological advancements in open pit mines, underground mines struggle to integrate the latest technologies, such as reliable wireless communication infrastructures, remote operation of mine vehicles, and rapid rescue of trapped miners [1]. Underground mines, intricate labyrinths spanning several kilometers with limited workspace (a few meters), interconnect various working zones. During a shift, miners engage in diverse tasks, including drilling, roof support maintenance, ore transportation, inspections of mine areas, and blasting. The unique features of underground mines, such as high humidity, temperature, hazardous gas concentration, dust, noise, and poor visibility, create a high-stress work environment, making it more challenging compared to conventional industrial settings [2]. The safety and health of miners are paramount in the mining industry, as the distinctive and demanding mining environments significantly impact both physical and mental well-being. A recent report highlighted that 5190 individuals died in the USA in a single year due to fatal injuries, with almost 2.8 million workers experiencing nonfatal injuries. The report emphasized that over 800,000 workers had to take time off work, averaging eight working days per year [3]. These occupational health and safety hazards underscore the magnitude of the problem in various industrial work environments. In underground mines, working in hazardous conditions is an inherent part of miners’ lives, and ensuring their safety and health remains a serious concern for mine management. Numerous data collection and health-related programs have been initiated to enhance health and safety for underground miners in the US [4]. However, these data collection methods are often periodic and limited to specific geographic distributions. Despite comprehensive periodic training on health, safety, and emergency preparedness, workplace injuries and accidents persist. Substantial research efforts are required to minimize safety and health risks, not only to improve miners’ quality of life but also to enhance overall operational productivity, as injuries or accidents can impede production activities. While many researchers monitored health monitoring, recent studies have explored ZigBee-based body area networks [5], context-aware sensing using wearable devices [6], and the role of wearable technology in mining environments [7]. However, these studies are often limited to simulations or lack networking architectures, insights into wireless signal characteristics in underground mines, and overall system functionality. In response to the urgent need for technological advancements and miners’ well-being in underground mines, our research aims to identify the best sensor for health sensing and predicting future health hazards among miners. Additionally, we seek to leverage the sensed health data for continuous location tracking of underground miners. Notably, industry 4.0 strategies can be used for securing underground mine excavations and tunnels [8,9,10]. Based on the conducted study by Poormirzaee et al. [11], internet of things (IoT) is an effective strategy for safe mining. Ali and Khan [12] considered the TODIM technique in green supplier selection.

The significance of this research lies in its pivotal contribution to advancing underground mine safety and health through the introduction of a Hybrid MEREC-CoCoSo System for the Selection of Best Sensor. The underground mining industry faces inherent challenges that necessitate innovative approaches to enhance safety protocols and mitigate health risks. By developing and implementing a hybrid system that combines the strengths of the MEREC (Multi-experts’ Evaluation based on Ratio and Criterion) methodology with the CoCoSo (Comprehensive Compromise Solution) method, this research aims to establish a robust decision-making framework for selecting the most effective sensor technologies. The selection of appropriate sensors is a critical aspect of ensuring the safety and well-being of miners, as well as the integrity of underground infrastructures. The proposed system not only addresses the complexities of decision-making in sensor selection but also integrates the expertise of multiple professionals, incorporating their occupational knowledge and experience. This holistic approach acknowledges the multidimensional nature of challenges in underground mines. The international significance of the research lies in its potential to set a new standard for decision-making processes in the selection of sensors for underground mining operations. As mining industries continue to evolve, embracing advanced technologies becomes imperative, making the identification of the best sensor crucial for preemptive safety measures. The outcomes of this research will not only impact the mining community by improving safety standards but will also contribute valuable insights to the broader field of decision science, where multi-criteria decision-making methodologies are continually evolving.

Despite significant advancements in mining technologies and safety practices, there exists a noticeable gap in the comprehensive integration of decision-making methodologies specifically tailored for the selection of sensors in underground mining operations. The current literature often lacks a unified approach that combines the strengths of Multi-experts’ Evaluation based on Ratio and Criterion (MEREC) with Comprehensive Compromise Solution (CoCoSo) methods. Existing studies primarily focus on individual aspects of underground mine safety and health, overlooking the development of a hybrid system that efficiently and effectively integrates the expertise of multiple professionals in selecting the best sensor technologies. The literature review reveals a scarcity of research addressing the intricate challenges associated with sensor selection in underground mining environments. Many studies focus on individual aspects such as sensor performance metrics, safety regulations, or technological advancements, but there is a noticeable void in methodologies that holistically consider multiple criteria and expert opinions in tandem. The integration of the MEREC and CoCoSo methods presents a unique opportunity to bridge this gap by providing a comprehensive decision-making framework that prioritizes both the technical specifications of sensors and the invaluable insights of experienced professionals. Furthermore, existing research often lacks a unified and standardized system for evaluating and ranking the performance of sensors in the context of underground mine safety. The absence of a well-defined, universally accepted approach hinders the development of consistent safety protocols across diverse mining operations. MEREC is a method employed for eliciting relative weights of criteria in a decision-making process. In the context of our study, MEREC serves as a pivotal component for determining the importance of each criterion in the sensor selection process. Similarly, the CoCoSo method, which stands for Concordance, Discordance, and Consistency, is introduced as a part of our proposed decision-making framework. CoCoSo provides a comprehensive approach to evaluating and ranking alternatives based on their performance against specified criteria. We have elaborated on the origins and foundational principles of CoCoSo, emphasizing its role as an established methodology in MCDM literature. This clarifies that CoCoSo is a recognized method, not introduced uniquely for this study. It is noteworthy that the hybridization refers to the synergistic integration of MEREC and CoCoSo, combining their respective strengths to create a more robust decision-making framework. This integration ensures a comprehensive and effective approach to sensor selection, leveraging the benefits of both methodologies. This research aims to address this gap by proposing a hybrid system that not only fills the void in existing methodologies but also establishes a standardized framework for sensor selection, thus contributing to the harmonization of safety practices in the underground mining sector. The present research seeks to not only fill the void in existing methodologies but also to establish a standardized framework. This framework, in turn, contributes significantly to the harmonization of safety practices within the underground mining sector. In alignment with this aim, the study endeavors to enhance the transparency, comprehensiveness, and efficiency of the sensor selection process, ultimately addressing the inherent challenges faced by mining professionals. The aims of the research are intricately connected to the chosen methodology, ensuring a coherent and targeted approach toward achieving the defined objectives. This revised formulation provides a clearer and more nuanced articulation of the research aims, emphasizing the role of the methodology in addressing the identified research gap.

## 2. Materials and Methods

**Figure 1:**
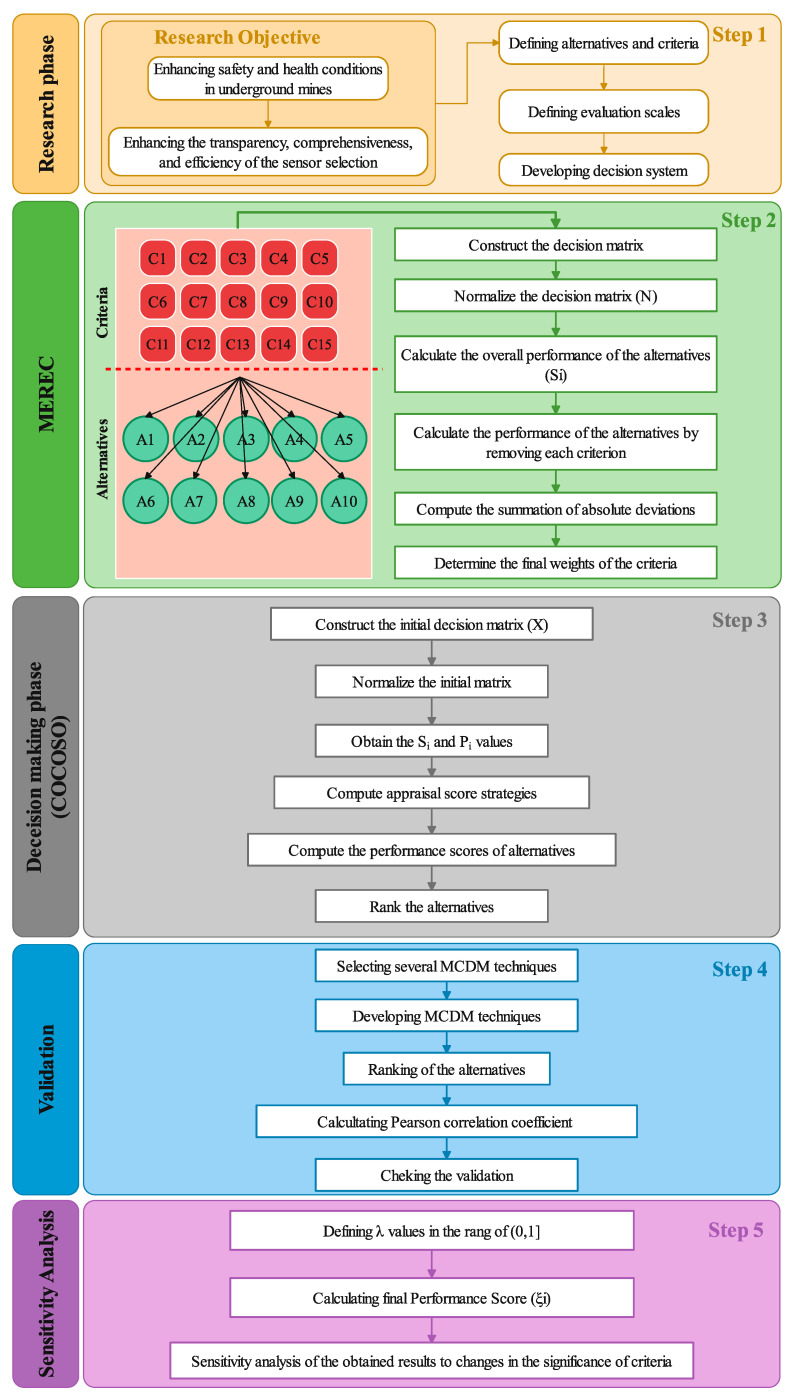
The proposed methodology for selection of appropriate sensor in underground mines.

The methodology employed in this research aims to address the critical aspect of enhancing safety and health conditions in underground mines through the selection of the most suitable sensor technologies. The aim of the research is to develop an effective decision-making framework for optimal sensor selection in underground mining operations [13,14]. To achieve this, the methodology employed integrates two key methods: the MEREC (Method for Eliciting Relative Weights) and combined Compromise Solution (CoCoSo) methodologies. These methods are strategically selected to enhance the transparency, comprehensiveness, and efficiency of the sensor selection process, addressing the specific challenges faced by mining professionals. To achieve this, we propose the integration of the MEREC and CoCoSO methods into a hybrid system. This approach seeks to leverage the strengths of both methods to provide a comprehensive and effective decision-making framework. The study flowchart for providing a hybrid decision framework, comprising five base phases, is presented in Figure 1. The initial stage is devoted to discerning sensors and the main criteria for safety and health in underground mines, grounded in a thorough expert knowledge and literature review. In this step, the decision system was formulated after the selection of ten sensors and 15 criteria. In the second stage, a MEREC technique was created to delineate relationships between identified factors and determine criteria weight. Following the precise formulation of the problem, the prioritization and ranking of sensors were carried out using the proposed MEREC-COCOSO framework in the third stage. In the fourth and fifth steps, the validation of the results and sensitivity analysis were conducted, respectively. A comprehensive elucidation of the MEREC technique and the phases of the CoCoSo system is expounded in the subsequent section.

### 2.1. Specifying Required Data

**Figure 2:**
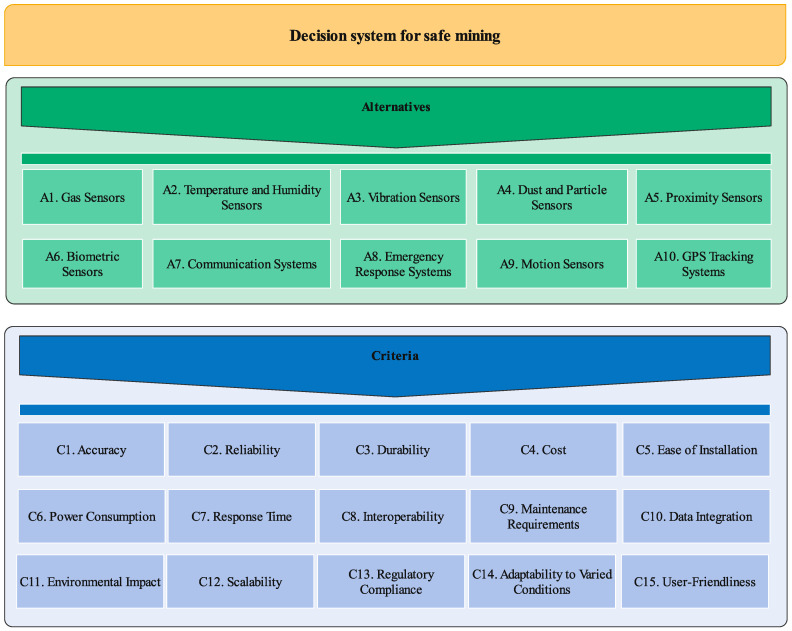
Components of decision-making system for evaluating mine excavation securing sensors.

The phase of securing underground mines and selecting the best sensor depends on various components such as installation and preparation costs, cost savings and income due to securing mines, response time, and power consumption, etc. In the first step of the study, decision-makers are requested and the initial investigations are conducted on the basis of literature reviews for specifying and determining the most critical criteria and alternatives. The formation of the problem was undertaken subsequent to the finalization of the predetermined set of criteria and securing sensors. As revealed in Figure 2, Accuracy (C1) [15], Reliability (C2) [16], Durability (C3) [17], Cost (C4) [11], Ease of Installation (C5) [18], Power Consumption (C6) [19], Response Time (C7) [20], Interoperability (C8) [21], Maintenance Requirements (C9) [22], Data Integration (C10) [23], Environmental Impact (C11) [24], Scalability (C12) [25], Regulatory Compliance (C13) [26], Adaptability to Varied Conditions (C14) [27], and User-Friendliness (C15) [28] are determined the most important criteria. Figure 2 illustrates the entirety of the decision system components examined in this current study. These components encompass 15 criteria and 10 securing underground mine plans identified as fundamental alternatives. The study integrates the most effective criteria for securing underground mines and securing sensors, identified by experts as paramount components, and introduces them for the first time. Dust and particle sensors (A4) play a vital role in ensuring health and safety in underground mines by monitoring and controlling the levels of airborne particulate matter. In the mining environment, the generation of dust and particles is a common occurrence, and prolonged exposure can lead to respiratory issues and other health hazards for workers. Dust and particle sensors are designed to detect and measure the concentration of these airborne particles, enabling mine operators to implement preventive measures and protect the workforce’s well-being. Dust and particle sensors can monitor particles of various sizes, including respirable dust that can penetrate deep into the lungs. This capability is crucial because different particle sizes may have different health implications, and regulatory standards often specify limits for specific size ranges. In underground mines, communication systems (A7) play a pivotal role in securing health and safety by facilitating effective and reliable communication among miners, rescue teams, and mine management. The unique challenges of the underground mining environment, including limited visibility, confined spaces, and the potential for emergencies, underscore the importance of robust communication systems. These systems enhance coordination, response times, and overall safety measures. Gas sensors (A1) [29,30] are designed to detect and monitor the levels of various gases that can pose severe threats to miners. The underground mining environment is prone to accumulating hazardous gases, which, if left undetected, can lead to life-threatening situations. Gas sensors are instrumental in identifying the presence of these gases and providing timely warnings, enabling miners to take necessary precautions. Gas sensors are specifically configured to detect the presence of hazardous gases commonly found in underground mines. These may include methane (CH_4_), carbon monoxide (CO), hydrogen sulfide (H_2_S), and various volatile organic compounds (VOCs). Early detection of these gases is crucial in preventing accidents and ensuring a safe working environment. GPS (Global Positioning System) tracking systems (A10) [31,32,33] in underground mines contribute significantly to securing health and safety by providing accurate location information for personnel, equipment, and vehicles within the mining environment. Despite the challenges of limited visibility and confined spaces in underground mines, GPS tracking systems enhance situational awareness, streamline operations, and play a crucial role in emergency response planning. GPS tracking systems in underground mines significantly secure health and safety by providing accurate location information for personnel, equipment, and vehicles within the mining environment. GPS tracking systems enable real-time tracking of miners’ locations, improving safety by allowing mine operators to monitor the whereabouts of personnel. In an emergency, such as a cave-in or gas leak, the system facilitates individuals’ swift identification and location, enabling faster and more effective rescue efforts. The tracking of mining equipment and vehicles is essential for optimizing operations and ensuring the safety of both personnel and machinery. GPS tracking systems provide the following:

- Insights into the movement and status of vehicles.
- Allowing for better coordination.
- Preventive maintenance planning.
- The identification of potential safety hazards.

GPS tracking systems are critical in coordinating response efforts during emergencies, such as accidents or evacuations. They provide real-time information on the location of personnel and resources, enabling rescue teams to navigate the underground environment more efficiently and respond promptly to incidents. GPS tracking systems can incorporate geofencing capabilities, allowing mine operators to define virtual boundaries and monitor when personnel or equipment enter or exit specific zones. This is particularly useful for identifying and preventing entry into hazardous areas, minimizing the risk of accidents and exposure to dangerous conditions. Proximity sensors (A5) [34,35] play a vital role in securing health and safety in underground mines by detecting the presence or proximity of objects, vehicles, or personnel. These sensors are designed to enhance situational awareness, prevent accidents, and contribute to the overall safety protocols in the confined and dynamic environment of underground mining operations. Proximity sensors are instrumental in preventing collisions between mining equipment, vehicles, and personnel. By detecting objects in close proximity, these sensors provide real-time warnings to operators, enabling them to take corrective actions and avoid accidents. This is especially crucial in the limited spaces of underground mines where visibility may be restricted. Underground mines often involve the use of heavy machinery and vehicles. Proximity sensors installed on equipment can detect obstacles or individuals in the immediate vicinity, reducing the risk of accidents such as collisions or crushing incidents. This enhances the safety of both equipment operators and other personnel working in the area. Proximity sensors contribute to pedestrian safety by detecting the presence of miners and workers in close proximity to moving equipment. Alarms or visual signals are triggered when someone is detected, alerting both the equipment operator and the pedestrian to the potential danger. This is particularly important in busy or congested areas of the mine. Proximity sensors can be integrated into systems that define safe zones and restricted areas within the mine. When personnel or equipment approach or enter restricted zones, the sensors trigger warnings, ensuring compliance with safety protocols and preventing unauthorized access to hazardous areas. In emergency situations, such as evacuations or rescue operations, proximity sensors provide critical information about the location and movement of personnel. This data assists emergency response teams in navigating the underground environment, locating individuals in distress, and executing rescue efforts more effectively. Temperature and humidity sensors (A2) [36,37] in underground mines play a crucial role in securing health and safety by monitoring environmental conditions. These sensors provide valuable data to mine operators, helping them manage potential risks associated with extreme temperatures, humidity levels, and other environmental factors. Maintaining optimal conditions is essential for the well-being of miners and the safe operation of equipment within the underground mining environment. Temperature and humidity sensors contribute to the well-being of underground miners by ensuring that working conditions remain within comfortable and safe ranges. Extreme temperatures or high humidity can lead to discomfort, fatigue, and increased stress on the human body. Monitoring these factors allows for adjustments in ventilation and other environmental controls to maintain a conducive and safe working atmosphere. In underground mines, especially those located in geographically warmer regions, excessive heat can pose a serious health risk to miners. Temperature sensors help identify areas where heat stress may be a concern, allowing for implementing preventive measures such as improved ventilation, hydration programs, and scheduled breaks to mitigate the risk of heat-related illnesses. Underground mining equipment often operates in challenging conditions, and temperature fluctuations can impact the performance and reliability of machinery. Temperature sensors help monitor equipment temperatures, enabling timely maintenance and preventing overheating or other issues that could compromise safety. Emergency response systems (A8) [38,39] in underground mines are essential components of safety protocols designed to secure the health and well-being of miners in the face of unforeseen incidents or emergencies. These systems are comprehensive frameworks that include communication tools, monitoring devices, evacuation procedures, and coordination strategies. The goal is to minimize the impact of emergencies, facilitate swift responses, and ensure the safety of personnel working in underground mines’ challenging and potentially hazardous environments. Vibration sensors (A3) [40] in underground mines play a vital role in securing health and safety by monitoring and assessing the levels of ground vibrations generated by mining activities. These sensors are instrumental in preventing structural damage, ensuring the stability of underground structures, and protecting the well-being of miners. Vibration sensors are employed to monitor ground stability in underground mines. Mining activities, such as blasting, drilling, or the operation of heavy machinery, can generate vibrations that may compromise the stability of surrounding rock formations. Continuous monitoring helps detect changes in ground vibrations, providing early warnings and allowing for proactive measures to maintain stability. Excessive vibrations in underground mines can contribute to rockfalls or cave-ins. Vibration sensors aid in identifying conditions that may lead to such events. Motion sensors (A9) [41] in underground mines play a pivotal role in securing health and safety by detecting movement and activity within the mining environment. These sensors contribute to various aspects of mine safety, including personnel tracking, equipment monitoring, and emergency response coordination. Motion sensors are utilized for tracking the movement of miners within the underground environment. By detecting the motion of individuals, these sensors contribute to real-time monitoring, helping mine operators keep track of personnel locations. This capability is crucial for emergency response, evacuation coordination, and ensuring the safety of miners during routine operations. In addition to personnel tracking, motion sensors are employed to monitor the movement of mining equipment and vehicles. This contributes to operational safety by preventing collisions, optimizing traffic flow, and providing data for the maintenance and management of equipment movement in confined underground spaces. Biometric sensors (A6) [42,43] in underground mines contribute to securing health and safety by employing unique biological characteristics to identify and monitor individuals. These sensors use physiological or behavioral attributes, such as fingerprints, iris patterns, or facial features, to ensure secure access control, track personnel, and enhance safety protocols within the underground mining environment. Biometric sensors are utilized for access control to restricted areas within the mine. By requiring biometric authentication, such as fingerprint or iris scans, these sensors ensure that only authorized personnel have access to specific zones. This helps prevent unauthorized entry into hazardous areas and enhances overall security. It can be concluded that applicable and new equipment in securing underground mines to provide health and safety can prove advantageous and profitable. Considering the applications of securing underground mine plans, it is recommended to embrace sensors that guarantee the health and safety of humans.

### 2.2. Adaptability of Criteria and Sensors to Varied Mining Contexts

The proposed system acknowledges the variations in environmental conditions commonly encountered in mining operations. For instance, underground mines pose challenges related to confined spaces, high humidity, and limited visibility. In contrast, open-pit mining operations may face exposure to varying weather conditions. The adaptability of the proposed system lies in its ability to customize criteria weights and prioritize sensors based on the specific environmental challenges of each mining context.

It should be mentioned that different types of mining, such as underground and open-pit, have distinct operational characteristics. This system recognizes the importance of tailoring criteria based on mining types. In underground mines, where confined spaces are prevalent, criteria such as “Ease of Installation” and “Power Consumption” may carry higher significance. In contrast, open-pit mines may prioritize criteria related to weather resistance, durability, and wide-area coverage [13,44].

Notably, the proposed system is designed to adapt to varying operational requirements within mining activities. For instance, during activities like drilling or blasting, real-time monitoring and immediate hazard detection become critical. Our system accommodates these considerations by allowing for dynamic adjustments in criteria importance, ensuring that safety measures align with specific operational demands.

### 2.3. Mining Safety and Proximity Analysis in GPS-Denied Areas

In underground mining environments, the reliance on GPS signals for proximity analysis poses significant challenges, particularly in areas where GPS signals may be denied or unreliable. This is a critical consideration, as underground mines often feature complex, unstructured terrains where traditional GPS may not be effective. To address this challenge, alternative technologies, such as LiDAR sensors, have gained prominence.

LiDAR (Light Detection and Ranging) sensors offer a promising solution for proximity analysis in GPS-denied areas within underground mines. Unlike GPS, LiDAR operates based on laser light reflections, allowing for accurate and real-time mapping of surroundings even in environments where GPS signals are obstructed. The application of LiDAR sensors in open pit mines has been explored in various studies, such as the work conducted by Balamurali and Mihankhah [45]. However, the applicability of LiDAR sensors in underground mines and GPS-denied scenarios warrants specific consideration.

Understanding the unique challenges of underground mining environments, where confined spaces and lack of direct line-of-sight may impede traditional proximity analysis methods, is crucial. LiDAR sensors can overcome these challenges by providing detailed 3D mapping and object detection capabilities. This ensures that proximity analysis is not solely dependent on GPS signals but extends to a more robust and versatile solution that can operate effectively in the complex, GPS-denied topography of underground mines [46,47].

### 2.4. MEREC

Ghorabaee et al. [48] introduced a method for determining weights based on the criterion removal effects, distinguishing itself from other methodologies that typically assess variance in alternative performance tied to criteria. Unlike conventional approaches, MEREC (Method for Elimination and Prioritization based on Enriched Characteristics) evaluates the impact of removing criteria to assign weights. The procedural steps for weight determination utilizing MEREC are detailed as following steps:

***Step 1: Formation of initial matrix.***

Formulate the decision matrix, represented as an *n* × *m* matrix, where *m* denotes the number of criterion and *n* signifies each alternative in the problem. The rows and columns involve performance values corresponding to the alternatives and criteria, denoted as *x_ij_*.

***Step 2: Normalization of the initial matrix.***

Normalize the decision matrix using the following formula:

(1) $$n_{ij}^{x}=\{\begin{array}{c} \frac{\underset{k}{min}\;x_{k_{j}}}{x_{ij}}\;\;\;\;\;\;\;if\;\;\;j\in B\\ \frac{x_{ij}}{\underset{k}{max}\;x_{k_{j}}}\;\;\;\;\;\;\;if\;\;\;j\in C \end{array}$$

where *B* stands the sets of beneficial criterion and *C* signifies the sets of non-beneficial (cost) criterion.

***Step 3: Calculation of overall performance of alternatives (S_i_).***

Compute the overall performance of alternatives (*S_i_*) using the following formula:

(2) Si=ln(1+(1m∑j|ln(nijx)|))

***Step 4: Determination of alternative performance ($S_{ij}^{\prime }$).***

Calculate alternative performance by removing the criteria:

(3) Sij′=ln(1+(1m∑kk≠j|ln(nikx)|))

***Step 5: Calculation of absolute deviation and E_j_.***

Compute the absolute deviation and sum it up to determine *E_j_*:

(4) $$E_{j}=\sum_{i}\left|S_{ij}^{\prime }-S_{i}\right|$$

***Step 6: Determination of each criterion weight (w_j_).***

Determine criterion weights using the formula:

(5) $$w_{j}=\frac{E_{j}}{\sum_{k}E_{k}}$$

In which, *w_j_* represents the weight determined for criteria for all MCDM techniques employed in this research.

### 2.5. CoCoSo

In 2019, Yazdani et al. [49] introduced the CoCoSo technique, a novel approach that amalgamates simple additive weighting with exponentially weighted product models. This method serves as a tool for conducting ranking or selection operations on various options. The CoCoSo method involves evaluating alternatives based on specific criteria, and its procedural steps are outlined as follows:

***Step 1: Formation of the initial decision matrix (X).***

In the first phase, a *m* × *n* matrix is organized as follows:

(6) $$X=\begin{array}{l} \begin{array}{ccccc} & C_{1} & C_{2} & \;\;\cdot \cdot \cdot & C_{4} \end{array}\\ \begin{array}{c} A_{1}\\ A_{2}\\ \vdots \\ A_{m} \end{array}{\left[\begin{array}{cccc} x_{11} & x_{12} & \cdot \cdot \cdot & x_{1n}\\ x_{21} & x_{22} & \cdot \cdot \cdot & x_{2n}\\ \vdots & \vdots & \ddots & \vdots \\ x_{m1} & x_{m2} & \cdot \cdot \cdot & x_{mn} \end{array}\right]}_{m\times n};\;\;\;\;\begin{array}{c} i=1,\;\;2,\;\;\ldots ,\;\;m\\ j=1,\;\;2,\;\;\ldots ,\;\;n \end{array} \end{array}$$

In which, *m* is alternatives and *n* is each criterion.

***Step 2: Preparation of the normalization matrix.***

To provide the normalization matrix (*Z*), the normalization of the initial decision matrix (*X*) is performed as Equation (7):

(7) $$Z=\begin{array}{l} \begin{array}{ccccc} & C_{1} & C_{2} & \;\;\cdot \cdot \cdot & C_{n} \end{array}\\ \begin{array}{c} A_{1}\\ A_{2}\\ \vdots \\ A_{m} \end{array}{\left[\begin{array}{cccc} {\bar{\bar{z}}}_{11} & {\bar{\bar{z}}}_{12} & \cdot \cdot \cdot & {\bar{\bar{z}}}_{1n}\\ {\bar{\bar{z}}}_{21} & {\bar{\bar{z}}}_{22} & \cdot \cdot \cdot & {\bar{\bar{z}}}_{2n}\\ \vdots & \vdots & \ddots & \vdots \\ {\bar{\bar{z}}}_{m1} & {\bar{\bar{z}}}_{m2} & \cdot \cdot \cdot & {\bar{\bar{z}}}_{mn} \end{array}\right]}_{m\times n};\;\;\;\;\begin{array}{c} i=1,\;\;2,\;\;\ldots ,\;\;m\\ j=1,\;\;2,\;\;\ldots ,\;\;n \end{array} \end{array}$$

For normalizing the decision matrix, the benefit and cost criteria are normalized by Equations (8) and (9), respectively.

(8) $${\bar{\bar{z}}}_{ij}=\frac{x_{ij}-x_{ij}^{-}}{x_{ij}^{+}-x_{ij}^{-}}$$

(9) $${\bar{\bar{z}}}_{ij}=\frac{x_{ij}^{+}-x_{ij}}{x_{ij}^{+}-x_{ij}^{-}}$$

where, $x_{ij}^{+}$ and $x_{ij}^{+}$ can be specified as Equations (10) and (11).

(10) $$x_{ij}^{+}=\underset{1\leq i\leq n}{max}\left(x_{ij}\right)$$

(11) $$x_{ij}^{-}=\underset{1\leq i\leq n}{min}\left(x_{ij}\right)$$

***Step 3: Calculation of the S_i_ and P_i_ values.***

The determination of the *S_i_* value follows the grey relational generation methodology, whereas the *P_i_* value is ascertained based on the multiplicative property of Weighted Aggregates Sum Product Assessment (WASPAS). The *S_i_* and *P_i_* values are calculated as Equations (12) and (13), respectively:

(12) $$S_{i}=\sum_{j=1}^{n}\left(w_{j}\cdot {\bar{\bar{z}}}_{ij}\right)$$

(13) $$P_{i}=\sum_{j=1}^{n}{\left({\bar{\bar{z}}}_{ij}\right)}^{w_{j}}$$

***Step 4: Determination of the appraisal scores strategies.***

The alternative’s relative weights are aggregated as Equations (14) and (15):

(14) $$\xi_{ia}=\frac{P_{i}+S_{i}}{\sum_{i=1}^{n}\left(P_{i}+S_{i}\right)}$$

(15) $$\xi_{ib}=\frac{S_{i}}{\underset{i}{min}\;S_{i}}+\frac{P_{i}}{\underset{i}{min}\;P_{i}}$$

(16) $$\xi_{ic}=\frac{\lambda \left(S_{i}\right)+\left(1-\lambda \right)\left(P_{i}\right)}{\left(\lambda \underset{i}{max}\;S_{i}+\left(1-\lambda \right)\underset{i}{max}\;P_{i}\right)}\;;\;\;0\leq \lambda \leq 1$$

Generally, *λ* = 0.5 is determined.

***Step 5: Providing the performance scores of alternatives.***

This score is calculated as Equation (17):

(17) ξi=(ξia⋅ξib⋅ξic)13+13(ξia+ξib+ξic)

***Step 6: Rank the alternatives.***

In the final stage, the alternatives are arranged in order according to their performance scores, and the optimal choice is identified as the option with the most elevated performance score.

### 2.6. Hybrid MEREC-CoCoSo System

The MEREC method will be employed to elicit the relative weights of the criteria considered in the sensor selection process. Ten domain experts, well versed in underground mine safety and health, will be engaged to assign weights to each criterion using a structured questionnaire. The MEREC method ensures a systematic and transparent process for collecting expert opinions on the importance of different criteria. Upon obtaining the criteria weights through MEREC, a concordance matrix will be constructed based on expert opinions. The matrix will capture the degrees of concordance between pairs of sensor alternatives concerning each criterion. The experts will use a scale of 1 to 9 to express their preferences. Simultaneously, a discordance matrix will be developed to assess the degrees of disagreement among the selected sensors. The CoCoSo method will be employed to analyze the discordance, taking into account the expert evaluations. The discordance matrix will help identify situations where a significant difference in performance is observed among sensors. Concordance and discordance indices will be calculated for each alternative sensor based on the expert opinions. These indices will serve as the basis for determining the overall performance of each sensor in relation to the specified criteria. The final decision-making process will involve combining the results from MEREC and CoCoSo. The relative weights obtained from MEREC will be integrated with the concordance and discordance indices to formulate a hybrid decision-making model. This model will provide a robust framework for selecting the best sensor for underground mine safety and health.

## 3. Results

The role of safety and health sensors in underground mines plays a pivotal role in not only ensuring the well-being of workers but also in significantly reducing costs associated with accidents, injuries, and operational inefficiencies [50]. These sensors are designed to monitor various environmental factors and human activities, providing real-time data that enables proactive measures to be taken. First and foremost, safety sensors contribute to accident prevention by detecting hazardous conditions such as gas leaks, unstable ground conditions, or equipment malfunctions. By promptly identifying potential risks, these sensors allow for immediate intervention, preventing accidents that could lead to injuries, damage to equipment, or even loss of life. This proactive approach not only safeguards the workforce but also minimizes the financial burden associated with medical expenses, compensation claims, and equipment replacement. Furthermore, safety and health sensors enhance overall operational efficiency in underground mines. By continuously monitoring equipment performance, these sensors can identify potential breakdowns or maintenance needs before they escalate into major issues. This proactive maintenance approach reduces downtime and prevents costly emergency repairs. Additionally, the real-time data provided by these sensors allows for optimized resource allocation, ensuring that personnel and equipment are deployed efficiently. In the long run, the investment in safety and health sensors proves to be cost-effective for mining operations. The reduction in accidents and illnesses translates into lower insurance premiums and compliance with regulatory standards, avoiding fines and legal consequences. Moreover, the enhanced operational efficiency leads to increased productivity and profitability. Hence, the role of safety and health sensors in underground mines is indispensable for minimizing costs associated with accidents, injuries, and operational inefficiencies. By providing real-time data and enabling proactive measures, these sensors contribute not only to the well-being of the workforce but also to the financial sustainability of mining operations. As technology continues to advance, the integration of sophisticated sensors will likely play an even more significant role in transforming the safety and efficiency landscape of underground mining.

### 3.1. Calculation of Criteria Weights by MEREC

Regarding the established criteria and available alternatives, a MEREC is organized to determine the weights assigned to the criteria. The viewpoints of ten experts, comprising four university professors and six professionals well versed in mining, were deliberated during a convened session and conclusively incorporated into the MEREC during the pairwise comparison stages. Expert 1 (Engineer, 8 years), Expert 2 (Manager, ≥12 years), Expert 3 (Engineer, ≥17 years), Expert 4 (Engineer, ≥25 years), Expert 5 (Manager, 12 years), Expert 6 (Manager, ≥17 years), Expert 7 (Associate professor, ≥13 years), Expert 8 (Assistant professor, ≥15 years), Expert 9 (Associate professor, ≥18 years), and Expert 10 (Associate professor, ≥23 years). The team of experts adopted the importance of criteria (C1–C15) using a value in the range 1–9. Experts used the scale reported in Table 1 for decision making regarding the importance of criteria.

By applying the defined scale, the team of experts provided an initial matrix. In fact, ten matrices were achieved from experts’ opinions, which were aggregated to obtain an initial matrix. Table 2 shows the initial matrix of expert opinions. As described in step 2 of MEREC technique, the initial decision matrix is converted to a normalized decision matrix as shown in Table 3. In the next step, the overall performance of alternatives (*S_i_*) and alternative performance ($S_{ij}^{\prime }$) are computed based on Equations (2) and (3), respectively. The calculated *S_i_* and $S_{ij}^{\prime }$ are presented in Table 4 and Table 5, respectively. Finally, the absolute deviation and Ej are determined as illustrated in Table 6. In the last phase of MEREC calculations, Equation (5) is used to determine the weight of criteria.

**Figure 3:**
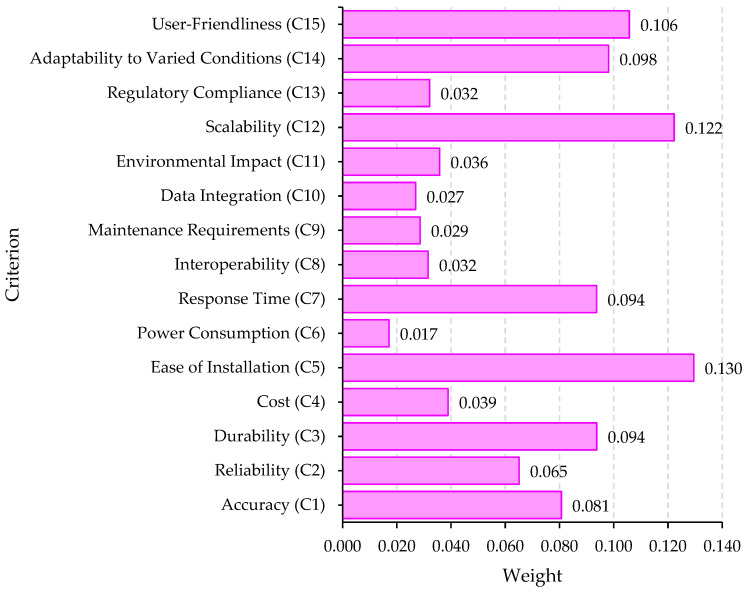
The obtained criteria’ weights based on MEREC technique.

The finalized weight for each criterion derived from the MEREC phase of the methodology is presented in Figure 3. As outlined in Table 7, the fifth criterion, “Ease of Installation”, carries the highest weight, amounting to 0.130, while the sixth criterion, “Power Consumption”, holds the lowest weight at 0.017. The results indicate a negligible difference of 0.113 between the weight of the most impactful criterion (0.130) and the least impactful (0.017). The prioritization of the 15 defined criteria, based on their importance determined through the MEREC method, is as follows: C5 > C12 > C15 > C14 > C3 > C7 > C1 > C2 > C4 > C11 > C13 > C8 > C9 > C10 > C6. This ranking underscores the substantial influence of all 15 criteria on the decision-making process for securing underground mines and selecting the optimal sensor.

### 3.2. MEREC–CoCoSo

This study introduces a decision-making framework designed to enhance the safety of underground excavations, with a specific focus on identifying the most suitable sensors for ensuring health and safety in mining operations. The novel MEREC-weighted CoCoSo approach, proposed in this research, serves as a robust MCDM technique. This method is systematically constructed by a panel of experts with significant occupational experience and expertise in the field. A team of experts has contributed valuable insights, providing two essential types of information: the weight assigned to criteria and the decision matrix, which incorporates the significance of all fifteen criteria within the alternatives. These decision-makers rigorously investigated a set of securing sensors against the backdrop of the fifteen identified criteria. It should be mentioned that each weight assigned to these criteria was derived during the MEREC phase of the methodology. The initial decision matrix, as outlined in Table 8, was established based on the first phase of the CoCoSo phase of the methodology. This comprehensive approach ensures a thorough evaluation and selection process for identifying the optimal sensors to enhance safety in underground mining operations.

Utilizing phase two of the CoCoSo method, specifically Equations (8)–(11) for benefit and cost criteria, the initial decision matrix underwent normalization, resulting in Table 9. This table not only includes the normalized values of the decision matrix but also encompasses the significance of criteria and the optimization direction for each criterion. To illustrate the normalization process, an example is provided as follows:

$$\mathrm{for}\;\mathrm{benefit}\;\mathrm{criteria}:\;{\bar{\bar{z}}}_{9,12}=\frac{x_{9,12}-x_{9,12}^{-}}{x_{9,12}^{+}-x_{9,12}^{-}}=\frac{7-2}{9-2}=0.7143$$

$$\mathrm{for}\;\mathrm{cost}\;\mathrm{criteria}:\;{\bar{\bar{z}}}_{3,8}=\frac{x_{3,8}^{+}-x_{3,8}}{x_{3,8}^{+}-x_{3,8}^{-}}=\frac{9-6}{9-2}=0.4286$$

Subsequently, the determination of the sum of weighted comparability sequences (*S_i_*) and power-weighted comparability sequences (*P_i_*) is carried out using Equations (12) and (13), and the results are presented in Table 10 and Table 11. The aggregated appraisal scores, *ξ_ia_*, *ξ_ib_*, and *ξ_ic_*, are computed according to Equations (14)–(16) and detailed in Table 12. The outcomes of the MEREC–CoCoSo methodology are explicitly provided, and the ranking of securing sensors is accomplished based on their respective ranks. To calculate the performance scores of alternatives, the *ξ* values are specified using Equation (17). An illustrative example is offered for alternative A8 as follows:

$$\xi_{5a}=\frac{P_{5}+S_{5}}{\sum_{i=1}^{10}\left(P_{5}+S_{5}\right)}=\frac{0.8303+3.7505}{6.702+125.2344}=0.1105$$

$$\xi_{5b}=\frac{S_{5}}{\underset{5}{min}\;S_{5}}+\frac{P_{5}}{\underset{5}{min}\;P_{5}}=\frac{0.8303}{0.4491}+\frac{13.7505}{9.941}=3.232$$

$$\xi_{5c}=\frac{\lambda \left(S_{5}\right)+\left(1-\lambda \right)\left(P_{5}\right)}{\left(\lambda \underset{5}{max}\;S_{5}+\left(1-\lambda \right)\underset{5}{max}\;P_{5}\right)}\;=\frac{0.5\times \left(0.8303\right)+\left(1-0.5\right)\left(13.7505\right)}{\left(0.5\times 0.8355+\left(1-0.5\right)\times 14.5836\right)}=0.9456$$

ξ5=(ξ5a⋅ξ5b⋅ξ5c)13+13(ξ5a+ξ5b+ξ5c)     =(0.1105×3.232×0.9456)13+13(0.1105+3.232+0.9456)     =2.1258

**Figure 4:**
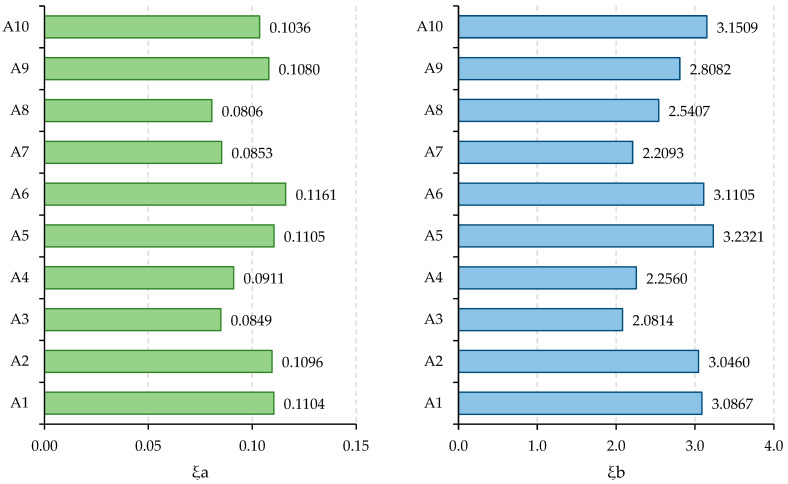

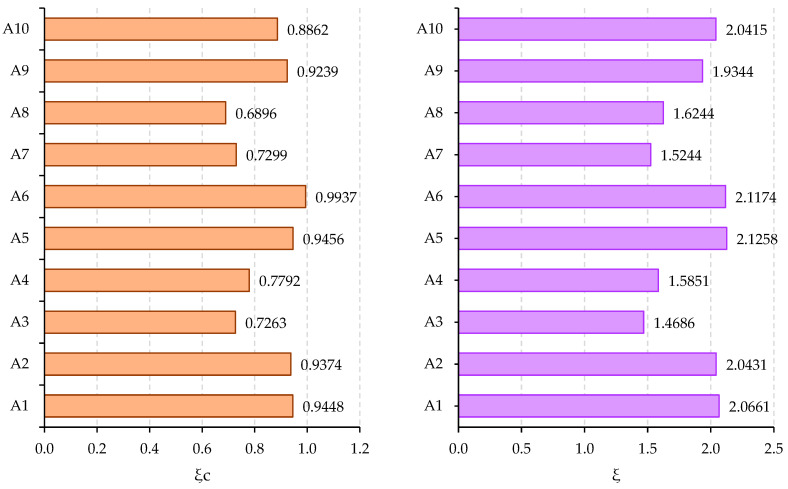
Prioritization order within each criterion.

The ranking of alternatives is as A5 > A6 > A1 > A2 > A10 > A9 > A8 > A4 > A7 > A3. According to the obtained results, the MEBEC-based CoCoSo model identified alternative A5, “proximity sensors”, as the optimal choice among various securing sensors for health and safe mining. Additionally, A6, “biometric sensors”, A1, “gas sensors”, and A2, “temperature and humidity sensors”, were respectively ranked as the second, third, and fourth most favorable sensors. The breakdown of rankings across the 15 criteria is presented in Table 12, with Figure 4 illustrating the distribution of rankings.

### 3.3. Validation of the Results

During this phase, the ranking of securing sensors was accomplished through analogous calculations utilizing various methodologies, including Vlse Kriterijumsk Optimizacija Kompromisno Resenje (VIKOR), Technique for Order Preference by Similarity to Ideal Solution (TOPSIS), TOmada de Decisao Interativa Multicriterio (TODIM), ELimination Et Choix Traduisant la REalite (ELECTRE), Complex Proportional Assessment (COPRAS), Evaluation Based on Distance from Average Solution (EDAS), and mulTi-noRmalization mUlti-distance aSsessment (TRUST). The aim was to corroborate the accepted results pertaining to the evaluation of progress. Table 13 presents the outcomes of VIKOR, TOPSIS, TODIM, ELECTRE, COPRAS, EDAS, and TRUST methods. Upon scrutinizing the results, notable similarities or inconsequential differences emerge between the results yielded by hybrid MEREC–CoCoSo and those of VIKOR, TOPSIS, TODIM, ELECTRE, COPRAS, EDAS, and TRUST. The comparison of our developed hybrid system with well-established methods such as VIKOR, TOPSIS, TODIM, ELECTRE, COPRAS, EDAS, and TRUST substantiates its effectiveness and the performance of the achieved results. This validation is further emphasized by the consistent first-place ranking of alternative A5 (proximity sensors) across various models. Consequently, the introduction of proximity sensors to mining excavations and tunneling projects is advocated as the optimal solution for safety and health working.

A correlation analysis was conducted to explore the concordance and disparity among the employed MCDM methods. The Pearson correlation coefficient, a well-known coefficient for assessing linear associations between two parameters, was selected to gauge the ranks’ correlation. The coefficient is computed using the following formula.

(18) $$C\left(X,Y\right)=\frac{\sum_{i=1}^{n}\left(x_{i}-\bar{x}\right)\left(y_{i}-\bar{y}\right)}{\sqrt{\sum_{i=1}^{n}\left(x_{i}-\bar{x}\right)\left(y_{i}-\bar{y}\right)}}$$

**Figure 5:**
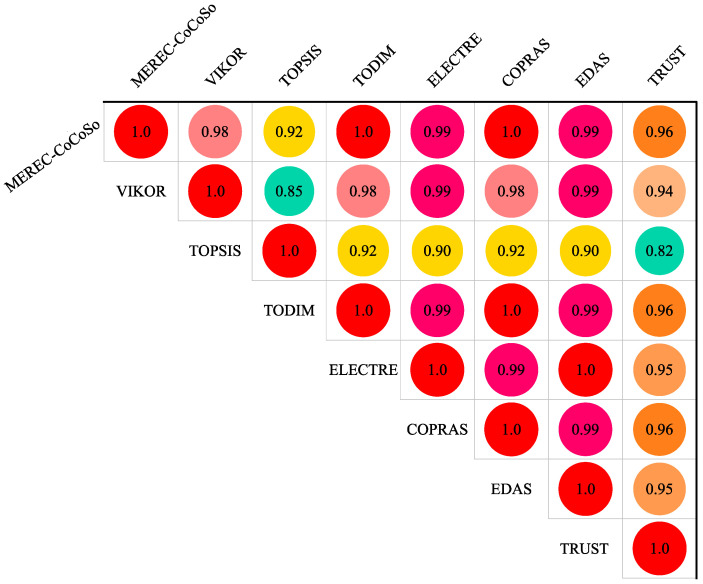
Pearson correlation coefficient of the ranking results.

Figure 5 illustrates the correlation matrix among the eight utilized techniques. In fact, the figure displays the Pearson correlation coefficients between the rankings generated by our proposed MEREC–CoCoSo model and those obtained from other well-established methods, namely VIKOR, TOPSIS, TODIM, ELECTRE, COPRAS, EDAS, and TRUST. The correlation coefficients range from −1 to 1, with 1 indicating a perfect positive correlation, 0 indicating no correlation, and −1 indicating a perfect negative correlation. High positive correlations suggest a consistent agreement between the rankings, while low correlations may indicate divergent outcomes. Any patterns or trends observed in the correlation matrix will be thoroughly discussed, contributing to a more insightful interpretation for the readers. Each technique exhibited a correlation of more than 85%, affirming the consistency of rankings derived in this study. The results indicated a high correlation among the selected eight MCDMs in identifying the best securing sensor. This suggests a likelihood that these methods would converge on the same optimal solution.

Generally, no conflicts were observed in the identification of the best and worst securing sensors by different MCDMs. However, due to variations in the methodology for calculating performance scores, discrepancies were noted in internal ranks. Nevertheless, since MCDMs are primarily employed to choose the superior compromise solution or prevent the worst, all eight methods were deemed reliable.

### 3.4. Sensitivity Analysis

**Figure 6:**
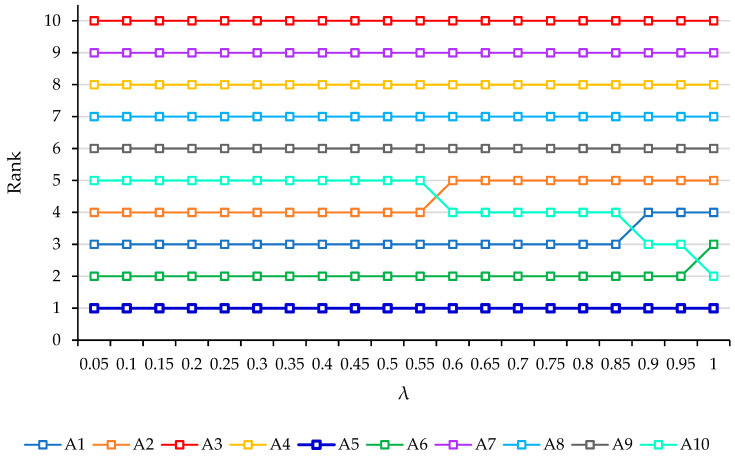
Ranking of the mine excavation securing strategies with different *λ* values.

In the final step of selecting the best alternative process and the decision system, a sensitivity analysis was performed to validate the ranking results. As part of this analysis, changes in the parameters of the principal developed system, MEREC-based CoCoSo, were introduced to confirm the decision-making process’s outcomes. The λ value in the CoCoSo technique was varied, and the final performance score (ξi) was calculated for result comparison. Various *λ* values were chosen within the range of (0, 1), as detailed in Table 14. The sensitivity test involved calculating the ranking of mine excavation securing strategies in each scenario, as illustrated in Figure 6. The results of the sensitivity test indicated the robustness of the CoCoSo model’s performance across different λ values. It can be inferred that the optimal securing strategy for safe and healthy mining, identified by the COCOSO model (alternative A5, “proximity sensors”), consistently maintained its optimal position. Consequently, the developed model demonstrated satisfactory performance and capability, rendering it applicable to similar real-world problems.

## 4. Conclusions

This research presents a pioneering approach to enhancing safety and health conditions in underground mines through the development of a hybrid MEREC–CoCoSo system for optimal sensor selection. The integration of the MEREC and CoCoSo methods, as outlined in the proposed decision-making framework, provides a robust and comprehensive methodology for addressing the complex considerations in securing underground mines. The study successfully discerns critical criteria and alternative sensors through expert knowledge and literature review, culminating in the prioritization of criteria based on the MEREC method. The results highlight the significance of “Ease of Installation” as the most influential criterion, emphasizing its pivotal role in the decision-making process. The subsequent ranking of alternatives identifies proximity sensors as the optimal choice for ensuring health and safety in mining operations. This conclusion is reinforced through comparisons with established methodologies, showcasing the reliability and consistency of the proposed MEREC–CoCoSo model. Furthermore, the research contributes to the field by conducting a thorough examination of MCDM methods, affirming the model’s reliability through consistent rankings. The validation process, including sensitivity analysis, reinforces the robustness of the COCOSO model in different scenarios, underscoring its applicability to real-world problems. In conclusion, the hybrid MEREC–CoCoSo system emerges as a powerful tool for decision-makers in the mining industry, providing a structured and effective approach to sensor selection that prioritizes safety and health. The identification of proximity sensors as the optimal choice signifies a valuable contribution to the advancement of underground mine safety. This research sets the stage for future developments in sensor technology and decision-making methodologies, with the potential to significantly impact safety practices in mining and related industries.

## Figures and Tables

**Table 1:** The used scale for determination of criteria importance [51,52,53,54].

Crisp Number	Linguistic Variable	Scale
1	Very Very Low	VVL
2	Very Low	VL
3	Relatively Low	RL
4	Low	L
5	Medium	M
6	High	H
7	Relatively High	RH
8	Very High	VH
9	Very Very High	VVH

**Table 2:** The initial matrix obtained from experts’ opinions.

Initial Matrix	C1	C2	C3	C4	C5	C6	C7	C8	C9	C10	C11	C12	C13	C14	C15
A1	RL	VVH	H	L	VH	RH	VVH	VL	H	VH	L	VH	RH	VH	H
A2	M	VH	RH	RH	VVH	M	VVH	VVH	RH	RH	RH	VH	RH	VVH	H
A3	RH	RH	VH	M	RH	RH	RL	H	H	RH	H	VL	M	RH	VL
A4	H	RH	RL	M	L	H	RH	H	RH	H	RH	RH	M	RH	M
A5	VH	H	VVH	VH	VVH	VH	VVH	VH	VH	VVH	M	VVH	VH	VVH	H
A6	RH	VVH	VH	RH	VH	RH	L	VH	H	VVH	RH	VVH	VH	VVH	M
A7	RH	L	VH	VH	RH	RH	VVH	VVH	RH	RH	RH	VL	RH	RL	RH
A8	H	VVH	VVH	VVH	VL	RH	VVH	VVH	VVH	VVH	VVH	VVH	VVH	VVH	RH
A9	RH	RH	RH	M	RH	RH	RH	H	H	RH	H	RH	M	RH	M
A10	VH	VVH	VH	RH	VVH	VH	VVH	VVH	VH	VVH	VH	VVH	VH	VVH	H

**Table 3:** The normalized decision matrix.

Normalized Matrix	C1	C2	C3	C4	C5	C6	C7	C8	C9	C10	C11	C12	C13	C14	C15
A1	1	0.444	0.500	0.444	0.250	0.875	0.333	0.222	0.667	0.750	0.444	0.250	0.714	0.375	0.333
A2	0.600	0.500	0.429	0.778	0.222	0.625	0.333	1	0.778	0.857	0.778	0.250	0.714	0.333	0.333
A3	0.429	0.571	0.375	0.556	0.286	0.875	1	0.667	0.667	0.857	0.667	1	1	0.429	1
A4	0.500	0.571	1	0.556	0.500	0.750	0.429	0.667	0.778	1	0.778	0.286	1	0.429	0.400
A5	0.375	0.667	0.333	0.889	0.222	1	0.333	0.889	0.889	0.667	0.556	0.222	0.625	0.333	0.333
A6	0.429	0.444	0.375	0.778	0.250	0.875	0.750	0.889	0.667	0.667	0.778	0.222	0.625	0.333	0.400
A7	0.429	1	0.375	0.889	0.286	0.875	0.333	1	0.778	0.857	0.778	1	0.714	1	0.286
A8	0.500	0.444	0.333	1	1	0.875	0.333	1	1	0.667	1	0.222	0.556	0.333	0.286
A9	0.429	0.571	0.429	0.556	0.286	0.875	0.429	0.667	0.667	0.857	0.667	0.286	1	0.429	0.400
A10	0.375	0.444	0.375	0.778	0.222	1	0.333	1	0.889	0.667	0.889	0.222	0.625	0.333	0.333

**Table 4:** Calculated overall performance of alternatives (*S_i_*).

ln (x)	C1	C2	C3	C4	C5	C6	C7	C8	C9	C10	C11	C12	C13	C14	C15	Sum	Si
A1	0	0.811	0.693	0.811	1.386	0.134	1.099	1.504	0.405	0.288	0.811	1.386	0.336	0.981	1.099	11.744	0.578
A2	0.511	0.693	0.847	0.251	1.504	0.470	1.099	0	0.251	0.154	0.251	1.386	0.336	1.099	1.099	9.952	0.509
A3	0.847	0.560	0.981	0.588	1.253	0.134	0	0.405	0.405	0.154	0.405	0	0	0.847	0	6.580	0.364
A4	0.693	0.560	0	0.588	0.693	0.288	0.847	0.405	0.251	0	0.251	1.253	0	0.847	0.916	7.593	0.410
A5	0.981	0.405	1.099	0.118	1.504	0	1.099	0.118	0.118	0.405	0.588	1.504	0.470	1.099	1.099	10.606	0.535
A6	0.847	0.811	0.981	0.251	1.386	0.134	0.288	0.118	0.405	0.405	0.251	1.504	0.470	1.099	0.916	9.867	0.505
A7	0.847	0	0.981	0.118	1.253	0.134	1.099	0	0.251	0.154	0.251	0	0.336	0	1.253	6.677	0.368
A8	0.693	0.811	1.099	0	0	0.134	1.099	0	0	0.405	0	1.504	0.588	1.099	1.253	8.684	0.457
A9	0.847	0.560	0.847	0.588	1.253	0.134	0.847	0.405	0.405	0.154	0.405	1.253	0	0.847	0.916	9.462	0.489
A10	0.981	0.811	0.981	0.251	1.504	0	1.099	0	0.118	0.405	0.118	1.504	0.470	1.099	1.099	10.439	0.528

**Table 5:** Calculated alternative performance.

Alternative Performance	C1	C2	C3	C4	C5	C6	C7	C8	C9	C10	C11	C12	C13	C14	C15
A1	0.578	0.547	0.552	0.547	0.525	0.573	0.536	0.520	0.563	0.567	0.547	0.525	0.566	0.541	0.536
A2	0.488	0.481	0.474	0.499	0.447	0.490	0.464	0.509	0.499	0.503	0.499	0.452	0.495	0.464	0.464
A3	0.324	0.337	0.317	0.336	0.304	0.357	0.364	0.345	0.345	0.357	0.345	0.364	0.364	0.324	0.364
A4	0.378	0.385	0.410	0.383	0.378	0.397	0.371	0.391	0.398	0.410	0.398	0.353	0.410	0.371	0.368
A5	0.496	0.519	0.491	0.530	0.474	0.535	0.491	0.530	0.530	0.519	0.512	0.474	0.516	0.491	0.491
A6	0.471	0.472	0.465	0.495	0.448	0.500	0.494	0.501	0.489	0.489	0.495	0.443	0.486	0.460	0.468
A7	0.328	0.368	0.322	0.363	0.309	0.362	0.316	0.368	0.357	0.361	0.357	0.368	0.353	0.368	0.309
A8	0.427	0.422	0.409	0.457	0.457	0.451	0.409	0.457	0.457	0.439	0.457	0.391	0.432	0.409	0.402
A9	0.454	0.466	0.454	0.465	0.437	0.484	0.454	0.472	0.472	0.483	0.472	0.437	0.489	0.454	0.451
A10	0.489	0.496	0.489	0.518	0.467	0.528	0.484	0.528	0.524	0.512	0.524	0.467	0.510	0.484	0.484

**Table 6:** Calculated absolute deviation and *E_j_*.

Absolute Deviation	C1	C2	C3	C4	C5	C6	C7	C8	C9	C10	C11	C12	C13	C14	C15
A1	0	0.031	0.026	0.031	0.053	0.005	0.042	0.058	0.015	0.011	0.031	0.053	0.013	0.037	0.042
A2	0.021	0.028	0.035	0.010	0.062	0.019	0.045	0	0.010	0.006	0.010	0.057	0.014	0.045	0.045
A3	0.040	0.026	0.047	0.028	0.060	0.006	0	0.019	0.019	0.007	0.019	0	0	0.040	0
A4	0.031	0.025	0	0.026	0.031	0.013	0.038	0.018	0.011	0	0.011	0.057	0	0.038	0.041
A5	0.039	0.016	0.044	0.005	0.061	0	0.044	0.005	0.005	0.016	0.023	0.061	0.019	0.044	0.044
A6	0.035	0.033	0.040	0.010	0.057	0.005	0.012	0.005	0.016	0.016	0.010	0.062	0.019	0.045	0.038
A7	0.040	0	0.046	0.005	0.060	0.006	0.052	0	0.012	0.007	0.012	0	0.016	0	0.060
A8	0.030	0.035	0.047	0	0	0.006	0.047	0	0	0.017	0	0.066	0.025	0.047	0.054
A9	0.035	0.023	0.035	0.024	0.053	0.005	0.035	0.017	0.017	0.006	0.017	0.053	0	0.035	0.038
A10	0.039	0.032	0.039	0.010	0.061	0	0.044	0	0.005	0.016	0.005	0.061	0.019	0.044	0.044
*E_j_*	0.310	0.250	0.360	0.149	0.497	0.066	0.360	0.121	0.110	0.103	0.137	0.469	0.123	0.377	0.406

**Table 7:** Calculated weight of criteria and their rank.

Criteria	Weight (*w_j_*)	Rank
Accuracy (C1)	0.081	7
Reliability (C2)	0.065	8
Durability (C3)	0.094	5
Cost (C4)	0.039	9
Ease of Installation (C5)	0.130	1
Power Consumption (C6)	0.017	15
Response Time (C7)	0.094	6
Interoperability (C8)	0.032	12
Maintenance Requirements (C9)	0.029	13
Data Integration (C10)	0.027	14
Environmental Impact (C11)	0.036	10
Scalability (C12)	0.122	2
Regulatory Compliance (C13)	0.032	11
Adaptability to Varied Conditions (C14)	0.098	4
User-Friendliness (C15)	0.106	3

**Table 8:** The initial decision matrix.

weights of criteria	0.081	0.065	0.094	0.039	0.130	0.017	0.094	0.032	0.029	0.027	0.036	0.122	0.032	0.098	0.106
Criteria	C1	C2	C3	C4	C5	C6	C7	C8	C9	C10	C11	C12	C13	C14	C15
A1	3	9	6	4	8	7	9	2	6	8	4	8	7	8	6
A2	5	8	7	7	9	5	9	9	7	7	7	8	7	9	6
A3	7	7	8	5	7	7	3	6	6	7	6	2	5	7	2
A4	6	7	3	5	4	6	7	6	7	6	7	7	5	7	5
A5	8	6	9	8	9	8	9	8	8	9	5	9	8	9	6
A6	7	9	8	7	8	7	4	8	6	9	7	9	8	9	5
A7	7	4	8	8	7	7	9	9	7	7	7	2	7	3	7
A8	6	9	9	9	2	7	9	9	9	9	9	9	9	9	7
A9	7	7	7	5	7	7	7	6	6	7	6	7	5	7	5
A10	8	9	8	7	9	8	9	9	8	9	8	9	8	9	6

**Table 9:** Normalized decision matrix.

Normalized Matrix	C1	C2	C3	C4	C5	C6	C7	C8	C9	C10	C11	C12	C13	C14	C15
A1	0	1	0.500	1	0.857	0.333	1	1	1	0.667	1	0.857	0.500	0.833	0.800
A2	0.400	0.800	0.667	0.400	1	1	1	0	0.667	0.333	0.400	0.857	0.500	1	0.800
A3	0.800	0.600	0.833	0.800	0.714	0.333	0	0.429	1	0.333	0.600	0	0	0.667	0
A4	0.600	0.600	0	0.800	0.286	0.667	0.667	0.429	0.667	0	0.400	0.714	0	0.667	0.600
A5	1	0.400	1	0.200	1	0	1	0.143	0.333	1	0.800	1	0.750	1	0.800
A6	0.800	1	0.833	0.400	0.857	0.333	0.167	0.143	1	1	0.400	1	0.750	1	0.600
A7	0.800	0	0.833	0.200	0.714	0.333	1	0	0.667	0.333	0.400	0	0.500	0	1
A8	0.600	1	1	0	0	0.333	1	0	0	1	0	1	1	1	1
A9	0.800	0.600	0.667	0.800	0.714	0.333	0.667	0.429	1	0.333	0.600	0.714	0	0.667	0.600
A10	1	1	0.833	0.400	1	0	1	0	0.333	1	0.200	1	0.750	1	0.800

**Table 10:** Weighted comparability sequence and Si value.

	C1	C2	C3	C4	C5	C6	C7	C8	C9	C10	C11	C12	C13	C14	C15	Si
A1	0	0.0651	0.0469	0.0389	0.1111	0.0057	0.0937	0.0315	0.0286	0.0180	0.0358	0.1048	0.0161	0.0818	0.0846	0.7625
A2	0.0323	0.0521	0.0625	0.0156	0.1296	0.0171	0.0937	0	0.0190	0.0090	0.0143	0.1048	0.0161	0.0981	0.0846	0.7488
A3	0.0646	0.0391	0.0781	0.0311	0.0925	0.0057	0	0.0135	0.0286	0.0090	0.0215	0	0	0.0654	0	0.4491
A4	0.0484	0.0391	0	0.0311	0.0370	0.0114	0.0625	0.0135	0.0190	0	0.0143	0.0874	0	0.0654	0.0635	0.4926
A5	0.0807	0.0260	0.0937	0.0078	0.1296	0	0.0937	0.0045	0.0095	0.0269	0.0287	0.1223	0.0241	0.0981	0.0846	0.8303
A6	0.0646	0.0651	0.0781	0.0156	0.1111	0.0057	0.0156	0.0045	0.0286	0.0269	0.0143	0.1223	0.0241	0.0981	0.0635	0.7380
A7	0.0646	0	0.0781	0.0078	0.0925	0.0057	0.0937	0	0.0190	0.0090	0.0143	0	0.0161	0	0.1058	0.5066
A8	0.0484	0.0651	0.0937	0	0	0.0057	0.0937	0	0	0.0269	0	0.1223	0.0321	0.0981	0.1058	0.6919
A9	0.0646	0.0391	0.0625	0.0311	0.0925	0.0057	0.0625	0.0135	0.0286	0.0090	0.0215	0.0874	0	0.0654	0.0635	0.6467
A10	0.0807	0.0651	0.0781	0.0156	0.1296	0	0.0937	0	0.0095	0.0269	0.0072	0.1223	0.0241	0.0981	0.0846	0.8355

**Table 11:** Calculated Pi values.

	C1	C2	C3	C4	C5	C6	C7	C8	C9	C10	C11	C12	C13	C14	C15	Pi
A1	0	1	0.9371	1	0.9802	0.9814	1	1	1	0.9891	1	0.9813	0.9780	0.9823	0.9767	13.8061
A2	0.9287	0.9856	0.9627	0.9650	1	1	1	0	0.9885	0.9708	0.9677	0.9813	0.9780	1	0.9767	13.7050
A3	0.9822	0.9673	0.9831	0.9914	0.9573	0.9814	0	0.9736	1	0.9708	0.9819	0	0	0.9610	0	10.7499
A4	0.9596	0.9673	0	0.9914	0.8502	0.9931	0.9627	0.9736	0.9885	0	0.9677	0.9597	0	0.9610	0.9474	11.5222
A5	1	0.9421	1	0.9393	1	0	1	0.9405	0.9691	1	0.9920	1	0.9908	1	0.9767	13.7505
A6	0.9822	1	0.9831	0.9650	0.9802	0.9814	0.8455	0.9405	1	1	0.9677	1	0.9908	1	0.9474	14.5836
A7	0.9822	0	0.9831	0.9393	0.9573	0.9814	1	0	0.9885	0.9708	0.9677	0	0.9780	0	1	10.7482
A8	0.9596	1	1	0	0	0.9814	1	0	0	1	0	1	1	1	1	9.9410
A9	0.9822	0.9673	0.9627	0.9914	0.9573	0.9814	0.9627	0.9736	1	0.9708	0.9819	0.9597	0	0.9610	0.9474	13.5994
A10	1	1	0.9831	0.9650	1	0	1	0	0.9691	1	0.9440	1	0.9908	1	0.9767	12.8286

**Table 12:** Results of MEBEC–COCOSO technique.

Alternative	*ξ_a_*	Rank	*ξ_b_*	Rank	*ξ_c_*	Rank	*ξ*	Final Rank
A1	0.1104	3	3.0867	4	0.9448	3	2.0661	3
A2	0.1096	4	3.0460	5	0.9374	4	2.0431	4
A3	0.0849	9	2.0814	10	0.7263	9	1.4686	10
A4	0.0911	7	2.2560	8	0.7792	7	1.5851	8
A5	0.1105	2	3.2321	1	0.9456	2	2.1258	1
A6	0.1161	1	3.1105	3	0.9937	1	2.1174	2
A7	0.0853	8	2.2093	9	0.7299	8	1.5244	9
A8	0.0806	10	2.5407	7	0.6896	10	1.6244	7
A9	0.1080	5	2.8082	6	0.9239	5	1.9344	6
A10	0.1036	6	3.1509	2	0.8862	6	2.0415	5

**Table 13:** The accuracy of the proposed technique compared to other techniques.

Alternative	Proposed Technique	VIKOR	TOPSIS	TODIM	ELECTRE	COPRAS	EDAS	TRUST
A1	3	4	3	3	3	3	3	2
A2	4	3	7	4	4	4	4	3
A3	10	10	10	10	10	10	10	10
A4	8	8	8	8	8	8	8	8
A5	1	1	2	1	1	1	1	1
A6	2	2	1	2	2	2	2	4
A7	9	9	9	9	9	9	9	9
A8	7	6	6	7	6	7	6	7
A9	6	7	5	6	7	6	7	6
A10	5	5	4	5	5	5	5	5

**Table 14:** The effect of different λ values on final performance score (ξ_i_).

	Final Performance Score (ξ_i_)																			
Alternative	λ																			
	0.05	0.1	0.15	0.2	0.25	0.3	0.35	0.4	0.45	0.5	0.55	0.6	0.65	0.7	0.75	0.8	0.85	0.9	0.95	1
A1	2.07	2.07	2.07	2.07	2.07	2.07	2.07	2.07	2.07	2.07	2.07	2.07	2.07	2.06	2.06	2.06	2.06	2.06	2.06	2.05
A2	2.04	2.04	2.04	2.04	2.04	2.04	2.04	2.04	2.04	2.04	2.04	2.04	2.04	2.04	2.04	2.04	2.04	2.04	2.03	2.02
A3	1.47	1.47	1.47	1.47	1.47	1.47	1.47	1.47	1.47	1.47	1.47	1.47	1.46	1.46	1.46	1.45	1.45	1.44	1.41	1.36
A4	1.59	1.59	1.59	1.59	1.59	1.59	1.59	1.59	1.59	1.59	1.58	1.58	1.58	1.58	1.57	1.57	1.56	1.55	1.53	1.47
A5	2.12	2.12	2.12	2.12	2.12	2.12	2.13	2.13	2.13	2.13	2.13	2.13	2.13	2.13	2.13	2.13	2.13	2.13	2.14	2.15
A6	2.12	2.12	2.12	2.12	2.12	2.12	2.12	2.12	2.12	2.12	2.12	2.12	2.11	2.11	2.11	2.11	2.10	2.10	2.09	2.05
A7	1.53	1.53	1.53	1.53	1.53	1.53	1.53	1.53	1.53	1.52	1.52	1.52	1.52	1.52	1.52	1.51	1.51	1.50	1.49	1.45
A8	1.62	1.62	1.62	1.62	1.62	1.62	1.62	1.62	1.62	1.62	1.63	1.63	1.63	1.63	1.63	1.64	1.64	1.65	1.66	1.70
A9	1.94	1.94	1.94	1.94	1.94	1.94	1.94	1.94	1.94	1.93	1.93	1.93	1.93	1.93	1.93	1.92	1.92	1.91	1.89	1.85
A10	2.04	2.04	2.04	2.04	2.04	2.04	2.04	2.04	2.04	2.04	2.04	2.04	2.04	2.05	2.05	2.05	2.05	2.06	2.07	2.11

## Data Availability

The data presented in this study are available on request from the corresponding author.
